# Kaji-Ichigoside F1 and Rosamultin Protect Vascular Endothelial Cells against Hypoxia-Induced Apoptosis via the PI3K/AKT or ERK1/2 Signaling Pathway

**DOI:** 10.1155/2020/6837982

**Published:** 2020-04-12

**Authors:** Chaofeng Shi, Li Zhan, Yuqiang Wu, Zhengchao Li, Jianyu Li, Yaxiao Li, Jinxia Wei, Yongliang Zhang, Lingzhi Li

**Affiliations:** ^1^Department of Pharmacy, Logistics University of Chinese People's Armed Police Forces, Tianjin 300309, China; ^2^Medical Team, Zhoukou Detachment of Chinese People's Armed Police Forces, Henan 466000, China; ^3^Characteristic Medical Center of Chinese People's Armed Police Forces, Tianjin 300309, China; ^4^Tianjin Key Laboratory for Prevention and Control of Occupational and Environmental Hazard, Tianjin 300309, China

## Abstract

As a pair of differential isomers, Kaji-ichigoside F1 and Rosamultin are both pentacyclic triterpenoids isolated from the subterranean root of *Potentilla anserina* L., a plant used in folk medicine in western China as antihypoxia and anti-inflammatory treatments. We demonstrated that Kaji-ichigoside F1 and Rosamultin effectively prevented hypoxia-induced apoptosis in vascular endothelial cells. We established a hypoxia model, using EA.hy926 cells, to further explore the mechanisms. Hypoxia promoted the phosphorylation of AKT, ERK1/2, and NF-*κ*B. In hypoxic cells treated with Kaji-ichigoside F1, p-ERK1/2 and p-NF-*κ*B levels were increased, while the level of p-AKT was decreased. Treatment with Rosamultin promoted phosphorylation of ERK1/2, NF-*κ*B, and AKT in hypoxic cells. Following the addition of LY294002, the levels of p-AKT, p-ERK1/2, and p-NF-*κ*B decreased significantly. Addition of PD98059 resulted in reduced levels of p-ERK1/2 and p-NF-*κ*B, while p-AKT levels were increased. Pharmacodynamic analysis demonstrated that both LY294002 and PD98059 significantly inhibited the positive effects of Kaji-ichigoside F1 on cell viability during hypoxia, consistent with the results of hematoxylin-eosin (H&E) staining, DAPI staining, and flow cytometry. The antihypoxia effects of Rosamultin were remarkably inhibited by LY294002 but promoted by PD98059. In Kaji-ichigoside F1- and Rosamultin-treated cells, Bcl2 expression was significantly upregulated, while expression of Bax and cytochrome C and levels of cleaved caspase-9 and cleaved caspase-3 were reduced. Corresponding to pharmacodynamic analysis, LY294002 inhibited the regulatory effects of Kaji-ichigoside F1 and Rosamultin on the above molecules, while PD98059 inhibited the regulatory effects of Kaji-ichigoside F1 but enhanced the regulatory effects of Rosamultin. In conclusion, Kaji-ichigoside F1 protected vascular endothelial cells against hypoxia-induced apoptosis by activating the ERK1/2 signaling pathway, which positively regulated the NF-*κ*B signaling pathway and negatively regulated the PI3K/AKT signaling pathway. Rosamultin protected vascular endothelial cells against hypoxia-induced apoptosis by activating the PI3K/AKT signaling pathway and positively regulating ERK1/2 and NF-*κ*B signaling pathways.

## 1. Introduction

*Potentilla anserina* L., a medicinal herb widely distributed in western China, is commonly used as an antihypoxia and anti-inflammatory treatment [1–4]. In order to identify the active component or components of *Potentilla anserina* L., we documented that the n-butanol extract of *Potentilla anserina* L. had obvious protective effects on the ischemia myocardium in mice [5]. Similarly, our research confirmed that the n-butanol extract of *Potentilla anserina* L. alleviated the myocardial ischemia-reperfusion injury and inhibited myocardial apoptosis in rats [6]. In addition, we demonstrated that the n-butanol extract of *Potentilla anserina* L. protected primary hippocampal neurons against hypoxia-induced injury by inhibiting caspase cascade reaction [7]. Furthermore, we isolated Kaji-ichigoside F1 and Rosamultin from the n-butanol extract of *Potentilla anserina* L. Kaji-ichigoside F1 and Rosamultin are differential isomers and are both pentacyclic triterpenoids. Cho et al. demonstrated that Rosamultin had potential for use as a therapeutic agent for treatment of various disorders involving free radical reactions [8]. Park et al. found that Rosamultin had antioxidant properties that might contribute to its protective effect against bromobenzene-induced hepatotoxicity in rats [9]. Morikawa et al. isolated Kaji-ichigoside F1 and Rosamultin from the tuberous roots of *Potentilla anserina* L. and also demonstrated their hepatoprotective effects, both *in vitro and in vivo* [10]. Jung et al. extracted Kaji-ichigoside F1 and Rosamultin from the roots of Rosa rugosa and demonstrated the anti-inflammatory/antinociceptive action of these compounds in acetic acid-induced writhing and hot plate testing and in a carrageenan-induced paw edema model in mice and rats [11]. Our previous studies indicated that Rosamultin activated phosphoinositide 3-kinase (PI3K)/AKT signaling pathways and had potential as a treatment for hydrogen peroxide-induced oxidative stress injury through its antioxidant and antiapoptotic effects in H9c2 cardiomyocytes [12]. In addition, we demonstrated that Kaji-ichigoside F1 and Rosamultin could effectively resist hypoxia-induced apoptosis in vascular endothelial cells. However, the antiapoptotic mechanisms of these isomers remain unclear. There are two major apoptotic pathways: the mitochondrial apoptotic pathway and the death receptor-mediated pathway [13–16]. The mitochondrial apoptotic pathway has become a popular research topic in recent years. It participates in the regulation of apoptotic processes in many cell types under hypoxic conditions by releasing Bcl2-associated x protein (Bax) and cytochrome C (Cyt C) [17]. Hypoxia-induced mitochondrial apoptosis is regulated by PI3K/AKT, mitogen-activated protein kinase (MAPK), nuclear factor- (NF-) *κ*B, hypoxia-inducible factor 1 (HIF-1), and other signaling pathways. Recently, PI3K/AKT and MAPK signaling pathways have attracted much attention [18]. In hypoxic cellular conditions, PI3K/AKT and MAPK signaling pathways are activated, which can protect cells by regulating biological processes, such as apoptosis, proliferation, and differentiation [19]. Song et al. demonstrated that procollagen-lysine,2-oxoglutarate 5-dioxygenase 2 (PLOD2) could promote the proliferation, differentiation, and invasion of glioma cells by activating the PI3K/AKT signaling pathway in hypoxic environment [20]. Zhao and Zheng noted that insulin growth factor 1 (IGF-1) prevented hypoxia-induced apoptosis in neural stem cells by activating the PI3K/AKT signaling pathway [21]. Shao et al. confirmed that MYB protooncogene like 2 (MYBL2) could inhibit apoptosis of H9c2 cardiomyocytes during hypoxia by activating PI3K/AKT and NF-*κ*B signaling pathways [22]. In addition, the activation of PI3K/AKT signaling during hypoxia can promote the expression of HIF-1*α* in cells [23]. MAPK is a serine-threonine protein kinase, which plays a role in intracellular and extracellular signal transduction in various cell types and regulates many important biological processes, such as differentiation, proliferation, and apoptosis [24–27]. Extracellular regulated kinase 1/2 (ERK1/2) is an important member of the MAPK family. Activation of the ERK1/2 signaling pathway has antiapoptotic effects in an ischemic myocardium [28–32]. Cui et al. reported that hypoxia promoted inactivation of the adenosine A2a receptor by activating the ERK1/2 signaling pathway and thereby reducing apoptosis [33]. Activation of the ERK1/2 signaling pathway during hypoxia has also been shown to be involved in regulating the activation of the HIF-1 signaling pathway [34]. Hanafi et al. showed that ursodeoxycholic acid could alleviate cobalt chloride-induced damage to cardiomyocytes by activating ERK1/2 and PI3K/AKT signaling pathways [35]. Yang et al. demonstrated that IGF-1 could inhibit hypoxia-induced apoptosis of retinal ganglion cells via activation of ERK1/2 and PI3K/AKT signaling pathways [36]. In this study, we established a model of hypoxia using EA.hy926 cells and used a PI3K/AKT pathway inhibitor, LY294002, and an ERK1/2 signaling inhibitor, PD98059, to explore (a) the correlation between the antiapoptotic effects of Kaji-ichigoside F1 and Rosamultin and the PI3K/AKT and ERK1/2 signaling pathways, (b) the interaction between PI3K/AKT and ERK1/2 signaling pathways during hypoxia, and (c) the effects of PI3K/AKT and ERK1/2 signaling on NF-*κ*B pathways, in order to elucidate the antiapoptotic mechanisms of action of Kaji-ichigoside F1 and Rosamultin at the cellular level.

## 2. Materials and Methods

### 2.1. Materials and Reagents

**Figure 1:**
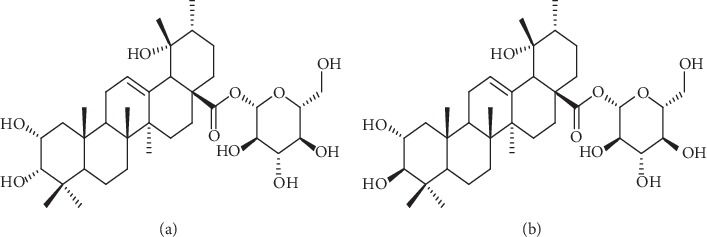
Chemical structure of Kaji-ichigoside F1 and Rosamultin. (a) Chemical structure of Kaji-ichigoside F1. Molecular formula: C_36_H_58_O_10_. (b) Chemical structure of Rosamultin. Molecular formula: C_36_H_58_O_10_.

Kaji-ichigoside F1 and Rosamultin (purity > 98%) were generated in our laboratory (Figure 1). LY294002 was obtained from MedChemExpress (New Jersey, USA); PD98059 was obtained from Selleckchem (Houston, USA). HyClone foetal bovine serum was obtained from Thermo Scientific (Massachusetts, USA), and DMEM cell culture medium was obtained from Solarbio (Beijing, China). Primary antibodies against p-ERK1/2, ERK1/2, p-AKT, AKT, p-NF-*κ*B, NF-*κ*B, Bcl-2, Bax, Cyt C, cleaved caspase-9, and cleaved caspase-3 were bought from Cell Signaling Technology (Boston, USA). Primary antibodies against *β*-actin were bought from Proteintech Group (Chicago, USA). The sequences of qPCR primers were as follows: caspase-9: F: CTAGTTTGCCCACACCCAGT and R: TGCTCAAAGATGTCGTCCAG; caspase-3: F: GTGGAGGCCGACTTCTTGTA and R: GTCGGCATACTGTTTCAGCA; Bax: F: TTTGCTTCAGGGTTTCATCC and R: TGAGACACTCGCTCAGCTTC; Bcl-2: F: GAGGATTGTGGCCTTCTTTG and R: GCCGGTTCAGGTACTCAGTC; Cyt C: F: CGTTCCTGCTGGTGATGTTG and R: GACCGATCAGACCATGCAGA; and actin: F: GCACTCTTCCAGCCTTCCTT and R: AATGCCAGGGTACATGGTGG.

### 2.2. Cell Culture and Treatment

EA.hy926 cells were purchased from the American Type Culture Collection (ATCC) and cultured in a cell incubator in a 37°C, 5% CO_2_ atmosphere. The cells were grown in a Petri dish. Adherent monolayers were dissociated using trypsin and either passaged or cryopreserved. Cells were cultured in 90% DMEM high glucose supplemented with 10% MRC foetal bovine serum. Cells were cryopreserved in 90% MRC foetal bovine serum supplemented with 10% DMSO. Cells were seeded at a concentration of 4 × 10^4^ cells/mL in cell culture medium for experiments. After 24 h, the culture medium was replaced with serum-free DMEM high glucose and the culture was allowed to expand for 24 h. A negative control group (normoxia) was established by replacing cell culture medium with fresh serum-free DMEM high glucose incubating for 2 h at 37°C in a 5% CO_2_ atmosphere before harvesting. For the hypoxia model, culture medium was aspirated off and replaced with the experimental reagents, diluted in D-Hanks solution. The untreated hypoxia control group was treated with the same volume of D-Hanks solution only. Treatment groups and the hypoxia control group were cultured in a three-gas incubator (37°C, 1% O_2_, 5% CO_2_, 94% N_2_) for 2 h to induce hypoxia. The cell groups were termed the normoxia control group, hypoxia model group, Kaji-ichigoside F1 (10^−13^ mol/L) treatment group, Rosamultin (10^−13^ mol/L) treatment group, and corresponding inhibitor intervention groups by the PI3K/AKT inhibitor LY294002 (10 *μ*mol/L) [37] or the ERK1/2 inhibitor PD98059 (10 *μ*mol/L) [38].

### 2.3. Cell Viability Detection by the Methyl-Thiazolyl-Tetrazolium (MTT) Assay

Cell viability was assessed by the MTT assay (Solarbio, Beijing, China). 20 *μ*L of MTT reagent (5 mg/mL) was added to cell culture wells in 200 *μ*L of culture medium and allowed to incubate for 4 h at 37°C. Plates were mixed, and 150 *μ*L of DMSO was added to each well to dissolve crystal formation. Absorbance of each culture well was read using a microplate reader (Sunrise, Wiesner Hager Möbel GmbH, Austria) at a wavelength of 490 nm.

### 2.4. Morphological Observation by H&E Staining

Cells were cultured in 6-well plates. After induction of hypoxia and drug treatment, the cell culture supernatant was discarded, and the cells were washed twice gently using precooled normal saline. Hematoxylin staining solution was added to the culture and allowed to incubate for 8 min. Cells were washed twice using tap water and then soaked in differentiation medium for 30 s, followed by eosin staining for 5 min. Stained cells were imaged using an inverted microscope (U-RFLT50, Olympus, Japan).

### 2.5. Cell Apoptosis Assay by DAPI Staining

After hypoxia induction and treatment, the cells were washed twice with 200 *μ*L of PBS. Cells were incubated for 15 minutes with a solution of 5 mL of DAPI dye (Solarbio, Beijing, China) in 500 mL PBS. Cells were incubated for 15 min in the dark, washed twice with PBS, and then observed using light microscopy (U-RFLT50, Olympus, Japan).

### 2.6. Detection of the Cell Apoptotic Rate by Flow Cytometry

Cells were digested, and cell concentration adjusted to 1 × 10^6^ cells/mL in PBS. Cells were stained with YF488-Annexin V and PI according to the manufacturer's instruction (Everbright Inc., California, USA). Flow cytometry (A00-1-1102, Beckman Coulter, USA) was used to detect the rate of apoptosis.

### 2.7. Western Blot Analysis

Cell lysates were harvested using RIPA buffer, according to the manufacturer's instructions (CWBIO, Beijing, China). Protein concentration was determined using the BCA protein assay according to the manufacturer's instructions (CWBIO, Beijing, China). p-ERK1/2, ERK1/2, p-AKT, AKT, p-NF-*κ*B, NF-*κ*B, Bcl-2, Bax, Cyt C, cleaved caspase-9, and cleaved caspase-3 proteins were detected using Western blot, as described previously [39]. In brief, samples were separated in 10% sodium dodecyl sulphate polyacrylamide gel electrophoresis (SDS-PAGE) and then transferred onto polyvinylidene fluoride (PVDF) membranes. After 1 h incubation in 5% (*w*/*v*) milk powder in PBS, the membranes were incubated with primary antibodies overnight, followed by incubation with secondary antibodies. Proteins were detected using WesternBright™ ECL (Advansta, California, USA) with a gel imaging analysis system (Tanon, Shanghai, China).

### 2.8. Detection of mRNA Levels by q-PCR

RNA was extracted using TRlzol, according to the manufacturer's instructions (CWBIO, Beijing, China). The extracted RNA was reverse transcribed to produce cDNA using a HiFiScript cDNA Synthesis Kit (CWBIO, Beijing, China). cDNA was then further amplified and detected using q-PCR with the UltraSYBR Mixture (CWBIO, Beijing, China), according to the manufacturer's instructions, and a CFX96 thermocycler (Bio-Rad, California, USA). The q-PCR parameters were predenaturation at 95°C for 10 min, denaturation at 95°C for 10 s, annealing at 60°C for 30 s, and extension at 72°C for 32 s. Samples were amplified over 45 cycles of denaturation, annealing, and extension. The relative quantitative 2^-*△△*Ct^ method was used for analysis, where △Ct = Ct (target gene) − Ct (internal reference gene) and *△△*Ct is obtained by subtraction of *△*Ct of the study sample from *△*Ct of the control sample. Fold change was determined using 2^-*△△*Ct^.

### 2.9. Statistical Analysis

Experimental data were presented as mean ± standard error of the mean (SEM). Statistical analysis was performed by ANOVA. *P* values less than 0.05 were considered statistically significant.

## 3. Results

### 3.1. Kaji-Ichigoside F1 and Rosamultin Regulated ERK1/2 and PI3K/AKT Signaling Pathways

**Figure 2:**
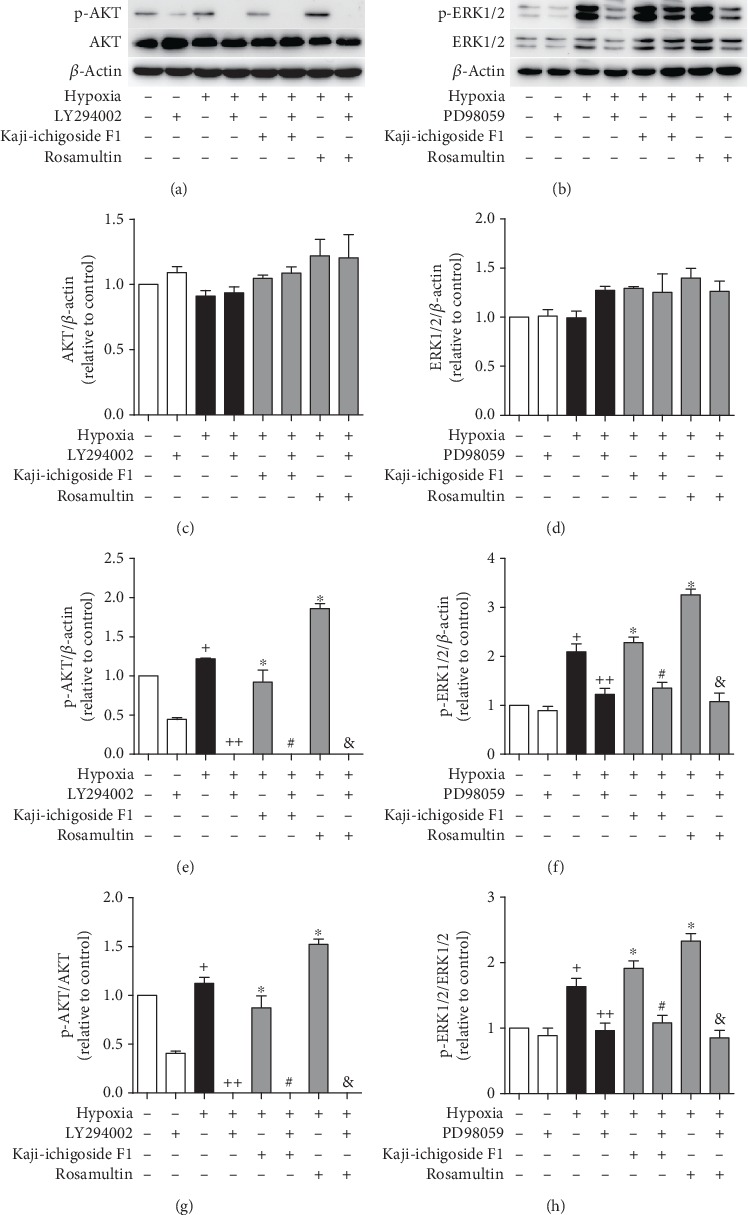
Kaji-ichigoside F1 activated the ERK1/2 signaling pathway and inhibited the PI3K/AKT signaling pathway, and Rosamultin activated PI3K/AKT and ERK1/2 signaling pathways. (a) Western blot showing the effects of Kaji-ichigoside F1, Rosamultin, and/or the PI3K/AKT inhibitor, LY294002, on p-AKT and AKT expressions. (b) Western blot showing the effects of Kaji-ichigoside F1, Rosamultin, and/or the ERK1/2 inhibitor, PD98059, on p-ERK1/2 and ERK1/2 expressions. (c) Quantitative analyses of AKT. (d) Quantitative analyses of ERK1/2. (e) Quantitative analyses of p-AKT. (f) Quantitative analyses of p-ERK1/2. (g) Quantitative analyses of p-AKT/AKT. (h) Quantitative analyses of p-ERK1/2/ERK1/2. Results are displayed as mean ± SEM (*n* = 3). ^+^*P* < 0.05 vs. normoxia control group, ^∗^^, ++^*P* < 0.05 vs. model group, ^#^*P* < 0.05 vs. Kaji-ichigoside F1 group. ^&^*P* < 0.05 vs. Rosamultin group.

Phosphorylation of AKT was significantly increased in the hypoxia model group compared to the normoxia control group (Figure 2). In hypoxic cells, Rosamultin treatment enhanced phosphorylation of AKT, while Kaji-ichigoside F1 treatment decreased AKT phosphorylation. LY294002 also significantly decreased the phosphorylation of AKT. There were no significant differences in protein expression of ERK1/2 among the different groups. However, exposure to hypoxia resulted in increased phosphorylation of ERK1/2, and compared with the hypoxia model group, both Kaji-ichigoside F1 treatment and Rosamultin treatment groups displayed enhanced phosphorylation of ERK1/2. PD98059-treated hypoxic cells showed significantly decreased phosphorylation of ERK1/2. These results indicated that Kaji-ichigoside F1 activated the ERK1/2 signaling pathway and inhibited the PI3K/AKT signaling pathway and that Rosamultin activated PI3K/AKT and ERK1/2 signaling pathways.

### 3.2. PI3K/AKT and ERK1/2 Signaling Pathways Regulated Each Other in Hypoxic Endothelial Cells

**Figure 3:**
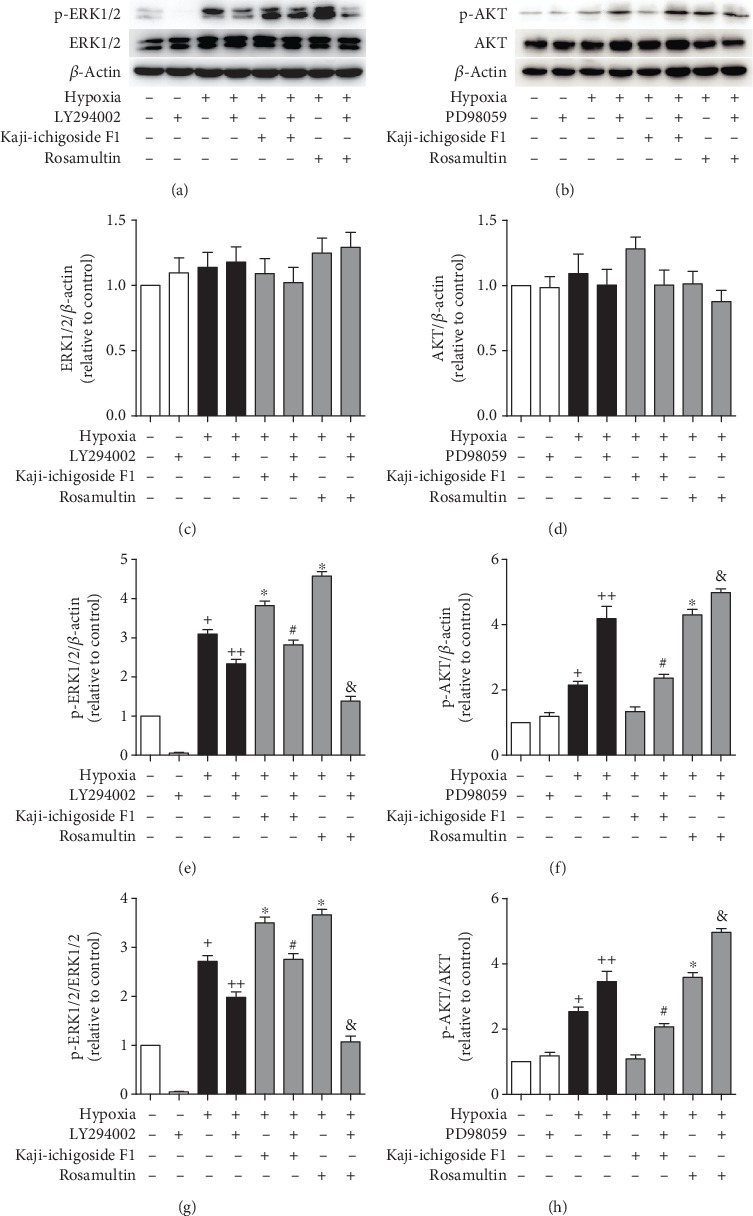
PI3K/AKT signaling positively regulated the ERK1/2 signaling pathway, while ERK1/2 signaling negatively regulated PI3K/AKT signaling. (a) Western blot showing the effects of Kaji-ichigoside F1, Rosamultin, and/or the PI3K/AKT inhibitor, LY294002, on p-ERK1/2 and ERK1/2 expressions. (b) Western blot showing the effects of Kaji-ichigoside F1, Rosamultin, and/or the ERK1/2 inhibitor, PD98059, on p-AKT and AKT expressions. (c) Quantitative analyses of ERK1/2. (d) Quantitative analyses of AKT. (e) Quantitative analyses of p-ERK1/2. (f) Quantitative analyses of p-AKT. (g) Quantitative analyses of p-ERK1/2/ERK1/2. (h) Quantitative analyses of p-AKT/AKT. Results are displayed as mean ± SEM (*n* = 3). ^+^*P* < 0.05 vs. normoxia control group, ^∗^^, ++^*P* < 0.05 vs. model group, ^#^*P* < 0.05 vs. Kaji-ichigoside F1 group. ^&^*P* < 0.05 vs. Rosamultin group.

ERK1/2 expression did not differ among the different treatment groups (Figure 3). In the hypoxia model group, the Kaji-ichigoside F1 group, and the Rosamultin group, LY294002 treatment blocked PI3K/AKT signaling and the expression of p-ERK1/2 was downregulated. In PD98059-treated cells, ERK1/2 signaling was also blocked; however, the expression of p-AKT was upregulated. These results indicated that PI3K/AKT signaling positively regulated the ERK1/2 signaling pathway and that ERK1/2 signaling negatively regulated the PI3K/AKT signaling pathway.

### 3.3. PI3K/AKT and ERK1/2 Signaling Pathways Positively Regulated NF-*κ*B Signaling

**Figure 4:**
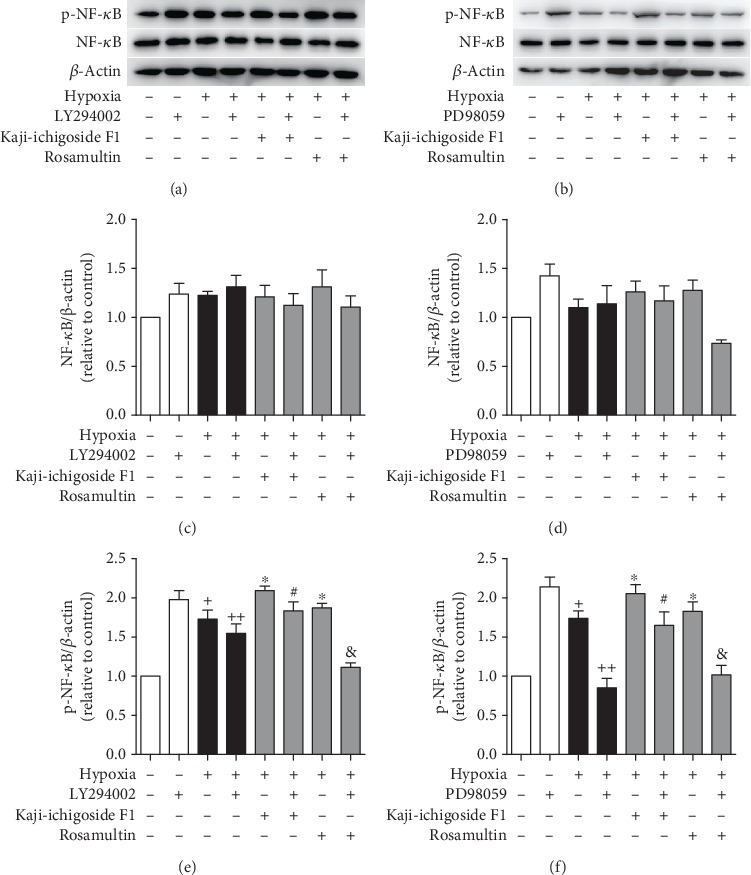
PI3K/AKT and ERK1/2 signaling pathways positively regulated NF-*κ*B signaling. (a) Western blot showing the effects of Kaji-ichigoside F1, Rosamultin, and/or the PI3K/AKT inhibitor, LY294002, on p-NF-*κ*B and NF-*κ*B. (b) Western blot showing the effects of Kaji-ichigoside F1, Rosamultin, and/or the ERK1/2 inhibitor, PD98059, on p-NF-*κ*B and NF-*κ*B. (c) Quantitative analyses of NF-*κ*B. (d) Quantitative analyses of NF-*κ*B. (e) Quantitative analyses of p-NF-*κ*B. (f) Quantitative analyses of p-NF-*κ*B. Results are displayed as mean ± SEM (*n* = 3). ^+^*P* < 0.05 vs. normoxia control group, ^∗^^, ++^*P* < 0.05 vs. model group, ^#^*P* < 0.05 vs. Kaji-ichigoside F1 group. ^&^*P* < 0.05 vs. Rosamultin group.

There was no significant difference in NF-*κ*B expression among the different groups (Figure 4). The level of p-NF-*κ*B in vascular endothelial cells subjected to hypoxia was significantly increased compared to the normoxia group. In Kaji-ichigoside F1 and Rosamultin treatment groups, p-NF-*κ*B was increased compared to that in the hypoxia model group. However, when either LY294002 or PD98059 was added to the Kaji-ichigoside F1 and Rosamultin treatment groups, the expression of p-NF-*κ*B downregulated. These results indicated that Kaji-ichigoside F1 and Rosamultin activated the NF-*κ*B signaling pathway and that NF-*κ*B signaling was positively regulated by PI3K/AKT and ERK1/2 signaling pathways.

### 3.4. Kaji-Ichigoside F1 and Rosamultin Mitigated Hypoxia-Induced Cell Damage by Activating ERK1/2 and Both PI3K/AKT and ERK1/2 Signaling Pathways, Respectively

**Figure 5:**
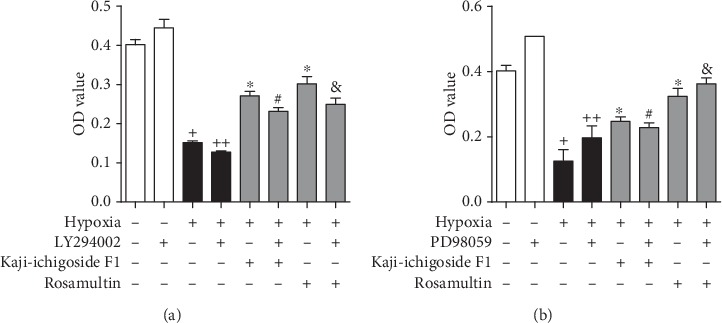
Kaji-ichigoside F1 enhanced cell viability via activation of ERK1/2 signaling, and Rosamultin enhanced cell viability via PI3K/AKT and ERK1/2 signaling pathways during hypoxia. (a) MTT assay showing the effects of Kaji-ichigoside F1, Rosamultin, and/or the PI3K/AKT inhibitor, LY294002, on cell viability. (b) MTT assay showing the effects of Kaji-ichigoside F1, Rosamultin, and/or the ERK1/2 inhibitor, PD98059, on cell viability. Results are displayed as mean ± SEM (*n* = 6). ^+^*P* < 0.05 vs. normoxia control group, ^∗^^, ++^*P* < 0.05 vs. model group, ^#^*P* < 0.05 vs. Kaji-ichigoside F1 group. ^&^*P* < 0.05 vs. Rosamultin group.

As shown in Figure 5, cell viability was significantly decreased in the hypoxia model group compared with the normoxia control group. Compared with the hypoxia model group, cell viability was significantly enhanced in the Kaji-ichigoside F1- and Rosamultin-treated cells. The addition of LY294002 resulted in decreased cell viability in the hypoxia model group and the Kaji-ichigoside F1- and Rosamultin-treated groups. Addition of PD98059 decreased cell viability in the Kaji-ichigoside F1-treated group but enhanced the cell viability in the hypoxia model and Rosamultin treatment groups.

**Figure 6:**
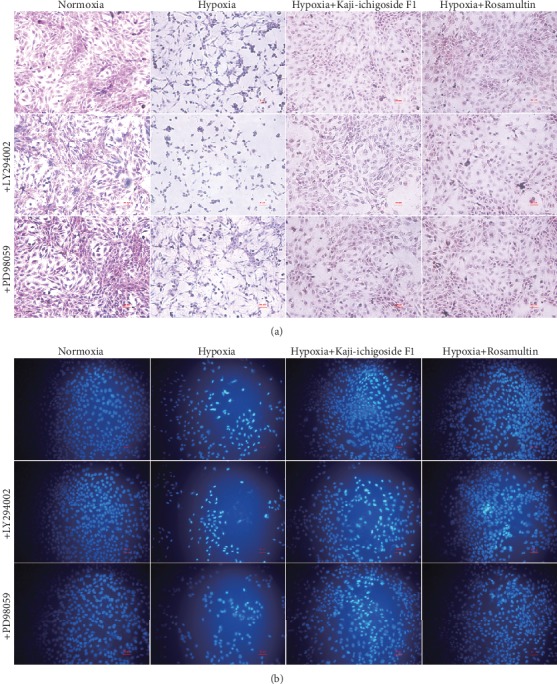
Kaji-ichigoside F1 reduced cell damage by activating ERK1/2 signaling, and Rosamultin reduced cell damage via PI3K/AKT and ERK1/2 signaling pathway activation during hypoxia. (a) H&E staining of EA.hy926 endothelial cells in each group. (b) DAPI staining of EA.hy926 endothelial cells in each group. Scale bar represents 50 *μ*m.

Consistent with these MTT assay results, H&E staining showed that cells in the normoxia control group adhered firmly, were densely distributed, and had normal cell morphology (Figure 6(a)). In the hypoxia model group, the cell adherence was poor with associated exfoliation/shedding, the distribution was sparse, and the cells appeared microcytic and unhealthy. The application of LY294002 increased cell exfoliation and aggravated the hypoxia-induced cell damage in the hypoxia model group and Kaji-ichigoside F1 and Rosamultin treatment groups. Addition of PD98059 also increased cell exfoliation and aggravated hypoxia-induced cell damage in the Kaji-ichigoside F1-treated group but decreased cell exfoliation and improved cell morphology in the hypoxia model group and the Rosamultin group.

### 3.5. Kaji-Ichigoside F1 and Rosamultin Inhibited Hypoxia-Induced Apoptosis by Activating ERK1/2 and Both PI3K/AKT and ERK1/2 Signaling Pathways, Respectively

**Figure 7:**
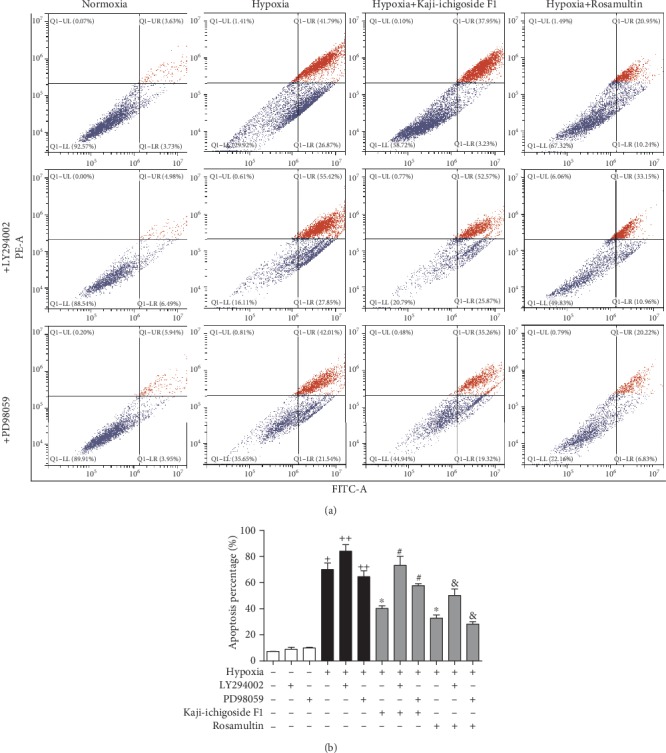
Kaji-ichigoside F1 decreased cell apoptotic rates via ERK1/2 signaling pathway activation, and Rosamultin decreased cell apoptotic rates via PI3K/AKT and ERK1/2 signaling pathway activation during hypoxia. (a) FACS analysis of apoptosis. (b) Cell apoptotic rate by FACS. The percentage of apoptotic cells is shown as mean ± SEM (*n* = 3). ^+^*P* < 0.05 vs. normoxia control group, ^∗^^, ++^*P* < 0.05 vs. model group, ^#^*P* < 0.05 vs. Kaji-ichigoside F1 group. ^&^*P* < 0.05 vs. Rosamultin group.

As indicated using DAPI staining, cells in the normoxia control group adhered firmly and were densely distributed with blue-white fluorescent nuclei (Figure 6(b)). Conversely, in the hypoxia model group, cell adherence was poor with associated shedding/exfoliation visible, distribution was sparse, nuclear concentration was more common, staining was brighter, and apoptotic cells were observed. Kaji-ichigoside F1 and Rosamultin treatment of cells subjected to hypoxia appeared to reduce cell exfoliation and the proportion of apoptotic cells. When LY294002 was added to the hypoxia model group, Kaji-ichigoside F1-treated group, or Rosamultin-treated groups, cell exfoliation was more widespread and a larger proportion of apoptotic cells were observed. However, when PD98059 was added to the hypoxia model group or the Rosamultin-treated group, cell exfoliation and the proportion of apoptotic cells decreased. Conversely, addition of PD98059 to the Kaji-ichigoside F1 group increased the percentage of apoptotic and exfoliated cells. Consistent with the above histochemistry results, flow cytometric analysis indicated that the apoptotic rate in cells from the normoxia control group was 7.36%, compared with the cells in the hypoxia model group, which had an apoptotic rate of 68.66% (Figure 7). The apoptotic rate increased to 83.27% when LY294002 was added to the hypoxia model group and decreased to 63.55% when PD98059 was added. The apoptotic rate of cells in the Kaji-ichigoside F1-treated group was 41.18%, which was lower than the hypoxia model group, but increased to 78.44% and 54.58% in Kaji-ichigoside F1-treated cells with the addition of LY294002 and PD98059, respectively. The apoptotic rate of cells in the Rosamultin-treated group was 31.19%, again, lower than the hypoxia model group. However, when LY294002 was added to hypoxic cells treated with Rosamultin, the apoptotic rate increased to 44.11% but decreased to 27.05% when PD98059 was added.

To investigate this potential relationship between the antimitochondrial apoptosis mechanisms of Kaji-ichigoside F1 and Rosamultin and PI3K/AKT or ERK1/2 signaling pathways, we investigated the effects of kinase inhibitors LY294002 and PD98059 on the expression of mitochondrial apoptosis-related genes.

**Figure 8:**
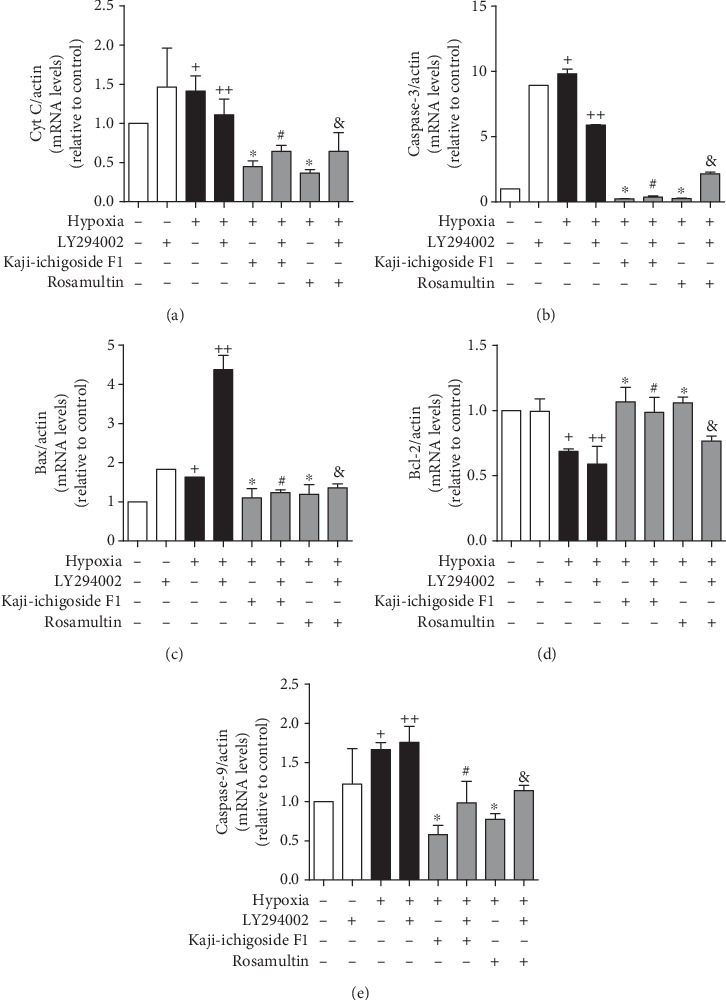
The results of q-PCR showed the antiapoptotic effects of Kaji-ichigoside F1 and Rosamultin, and Rosamultin inhibited hypoxia-induced apoptosis via the PI3K/AKT signaling pathway at the gene level. (a) Quantitative analyses of Cyt C mRNA expression. (b) Quantitative analyses of caspase-3 mRNA expression. (c) Quantitative analyses of Bax mRNA expression. (d) Quantitative analyses of Bcl-2 mRNA expression. (e) Quantitative analyses of caspase-9 mRNA expression. Results are displayed as mean ± SEM (*n* = 3). ^+^*P* < 0.05 vs. normoxia control group, ^∗^^, ++^*P* < 0.05 vs. model group, ^#^*P* < 0.05 vs. Kaji-ichigoside F1 group. ^&^*P* < 0.05 vs. Rosamultin group.

**Figure 9:**
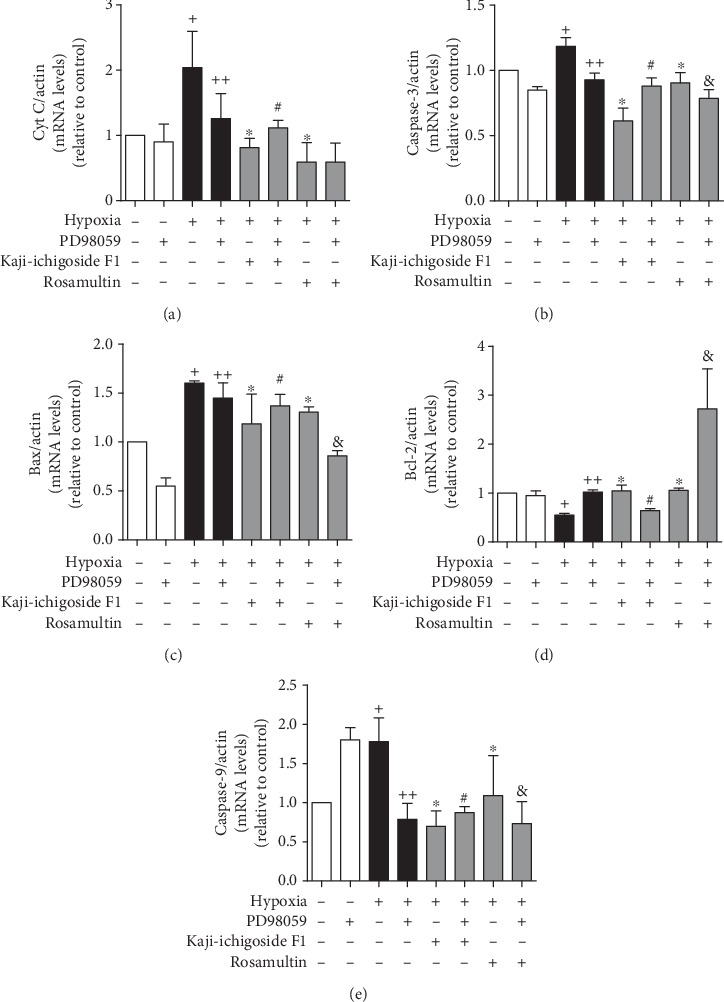
Kaji-ichigoside F1 and Rosamultin inhibited hypoxia-induced apoptosis by activating the ERK1/2 signaling pathway at the gene level. (a) Quantitative analyses of Cyt C mRNA expression. (b) Quantitative analyses of caspase-3 mRNA expression. (c) Quantitative analyses of Bax mRNA expression. (d) Quantitative analyses of Bcl-2 mRNA expression. (e) Quantitative analyses of caspase-9 mRNA expression. Results are displayed as mean ± SEM (*n* = 3). ^+^*P* < 0.05 vs. normoxia control group, ^∗^^, ++^*P* < 0.05 vs. model group, ^#^*P* < 0.05 vs. Kaji-ichigoside F1 group. ^&^*P* < 0.05 vs. Rosamultin group.

**Figure 10:**
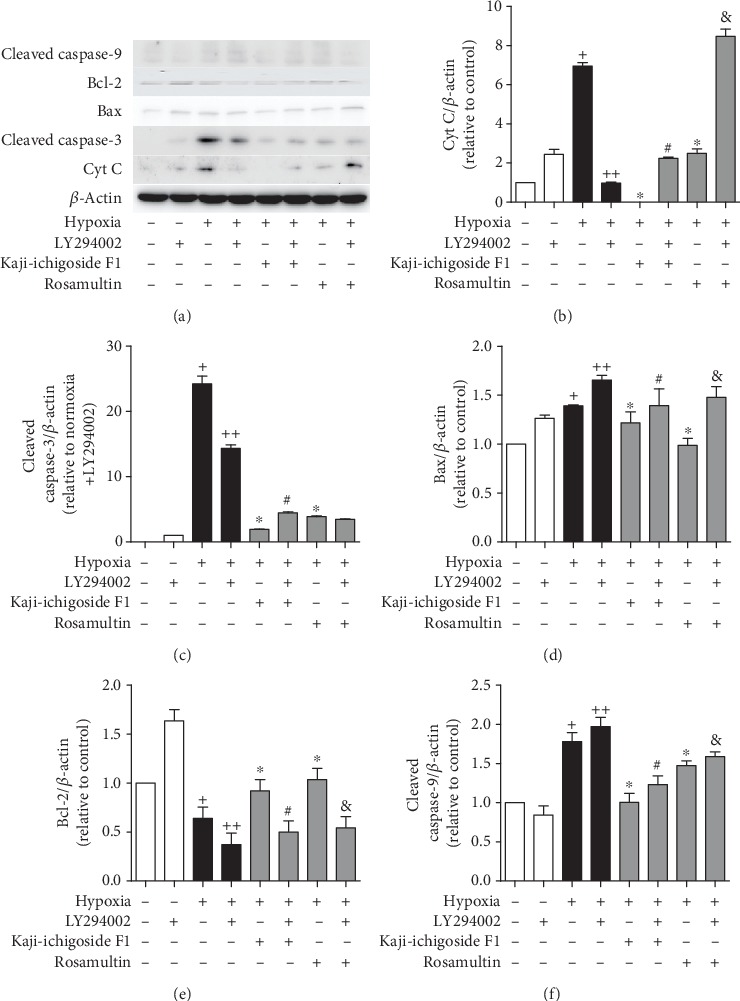
The results of Western blot showed the antiapoptotic effects of Kaji-ichigoside F1 and Rosamultin, and Rosamultin inhibited hypoxia-induced apoptosis via the PI3K/AKT signaling pathway at the protein level. (a) Western blot of Cyt C, cleaved caspase-3, Bax, Bcl-2, and cleaved caspase-9 in Kaji-ichigoside F1-, Rosamultin-, and/or, PI3K/AKT inhibitor LY294002-treated endothelial cells. (b) Quantitative analyses of Cyt C. (c) Quantitative analyses of cleaved caspase-3. (d) Quantitative analyses of Bax. (e) Quantitative analyses of Bcl-2. (f) Quantitative analyses of cleaved caspase-9. Results are displayed as mean ± SEM (*n* = 3). ^+^*P* < 0.05 vs. normoxia control group, ^∗^^, ++^*P* < 0.05 vs. model group, ^#^*P* < 0.05 vs. Kaji-ichigoside F1 group. ^&^*P* < 0.05 vs. Rosamultin group.

**Figure 11:**
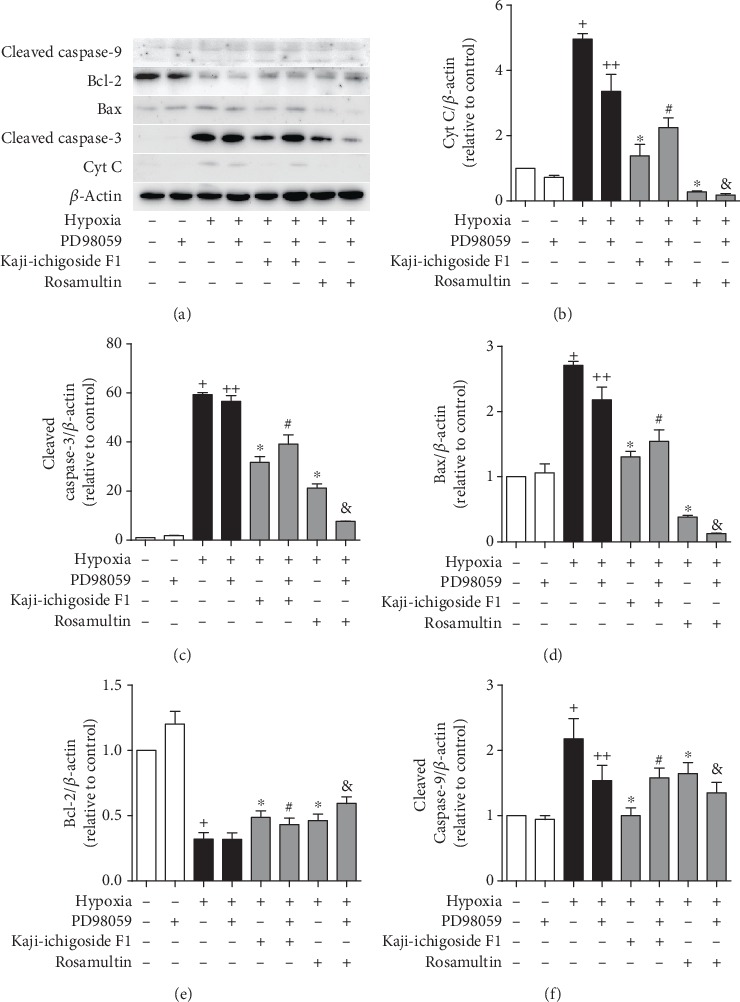
Kaji-ichigoside F1 and Rosamultin inhibited hypoxia-induced apoptosis by activating the ERK1/2 signaling pathway at the protein level. (a) Western blot of Cyt C, cleaved caspase-3, Bax, Bcl-2, and cleaved caspase-9 expression in Kaji-ichigoside F1-, Rosamultin-, and/or, ERK1/2 inhibitor PD98059-treated endothelial cells. (b) Quantitative analyses of Cyt C. (c) Quantitative analyses of cleaved caspase-3. (d) Quantitative analyses of Bax. (e) Quantitative analyses of Bcl-2. (f) Quantitative analyses of cleaved caspase-9. Results are displayed as mean ± SEM (*n* = 3). ^+^*P* < 0.05 vs. normoxia control group, ^∗^^, ++^*P* < 0.05 vs. model group, ^#^*P* < 0.05 vs. Kaji-ichigoside F1 group. ^&^*P* < 0.05 vs. Rosamultin group.

Compared with the normoxia control group, expression of B-cell lymphoma 2 (Bcl-2) mRNA in the hypoxia model group was downregulated, while Bax, Cyt C, caspase-9, and caspase-3 mRNA levels were upregulated (Figures 8 and 9). Kaji-ichigoside F1 and Rosamultin treatment effectively mitigated these changes in mRNA expression in response to hypoxia. In cells from the hypoxia model group and Kaji-ichigoside F1- and Rosamultin-treated groups, Bcl-2 mRNA expression was downregulated, while Bax, Cyt C, caspase-9, and caspase-3 mRNA expressions were upregulated following the addition of the PI3K/AKT inhibitor, LY294002. Addition of the MAPK inhibitor, PD98059, to cells in the Kaji-ichigoside F1-treated group also mitigated the effects of Kaji-ichigoside F1 on mitochondrial apoptosis-related gene expressions in response to hypoxia. However, inhibition by PD98059 in cells from the hypoxia model group and the Rosamultin-treated group resulted in upregulation of Bcl-2 mRNA level and downregulation of Bax, Cyt C, caspase-9, and caspase-3 mRNA expressions. Meanwhile, the expression levels of Bcl-2, Bax, Cyt C, cleaved caspase-9, and cleaved caspase-3 were consistent with the corresponding gene expressions (Figures 10 and 11).

## 4. Discussion

The expression of HIF-1*α* and phosphorylation of key molecules, such as ERK1/2, AKT, and NF-*κ*B, increase during hypoxia, indicating activation of protective signaling pathways including MAPK, PI3K/AKT, HIF-1, and NF-*κ*B [40–42]. These pathways participate in the regulation of mitochondrial apoptosis and effectively inhibit apoptosis [43]. Mitochondrial apoptosis starts from endogenous signals that lead to programmed cell death. Bcl-2 activates intracellular signals resulting in the release of Bax, which acts to alter the structure of the mitochondrial membrane and open the mitochondrial membrane permeability transition pore (MPTP). Cyt C and other proapoptotic molecules are then released into the cytoplasm via the MPTP. In the cytoplasm, Cyt C binds the apoptotic activator of cytoplasmic protein (Apaf-1) to enhance the polymerisation capacity of Apaf-1. Aggregates of Apaf-1/Cyt C can bind ATP/dATP and connect to the caspase recruitment area (CARD), thereby recruiting caspase-9 and initiating a caspase cascade reaction. Eventually, caspase-3 executes apoptosis [44–46].

**Figure 12:**
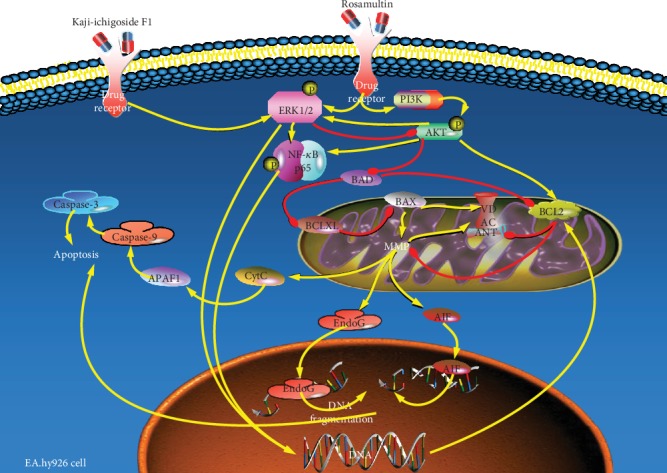
Schematic diagram depicting the cytoprotective effects of Kaji-ichigoside F1 and Rosamultin on hypoxia-induced apoptosis of EA.hy926 endothelial cells. Hypoxia causes mitochondrial apoptosis through activation of caspase-3. Rosamultin inhibits apoptosis induced by hypoxia by activating PI3K/AKT and ERK1/2 signaling pathways in EA.hy926 endothelial cells. Kaji-ichigoside F1 inhibits apoptosis via ERK1/2 signaling pathway activation. The PI3K/AKT signaling pathway positively regulates ERK1/2 and NF-*κ*B signaling pathways, while ERK1/2 signaling positively regulates NF-*κ*B signaling and negatively regulates the PI3K/AKT signaling pathway.

The relationship between signaling pathways, observed herein, was intricate. There was interaction between MAPK, PI3K/AKT, HIF-1, NF-*κ*B, and other signaling pathways [47]. This experiment confirmed that AKT phosphorylation promoted the phosphorylation of ERK1/2 and NF-*κ*B and that the PI3K/AKT pathway positively regulated ERK1/2 and NF-*κ*B signaling pathways. Our results also confirmed that ERK1/2 signaling negatively regulated the PI3K/AKT signaling pathway and positively regulated NF-*κ*B signaling, consistent with previously reported experimental results [34, 48–52]. In this study, the pharmacodynamic analyses were consistent with the results of q-PCR and Western blot, which showed that Kaji-ichigoside F1 and Rosamultin could inhibit mitochondrial apoptosis by altering the expression of mitochondrial apoptosis-related genes, including Bcl-2, Bax, Cyt C, caspase 9, and caspase 3. Pharmacodynamics, as well as gene and protein expression analyses, confirmed that the antiapoptotic effects of Rosamultin were related to the activation of PI3K/AKT and ERK1/2 signaling pathways in vascular endothelial cells. Conversely, while Kaji-ichigoside F1 inhibited mitochondrial apoptosis induced by hypoxia by activating the ERK1/2 signaling pathway, this ERK1/2 activation also acted to inhibit PI3K/AKT signaling. The relationship between these signaling pathways is shown in Figure 12. The PI3K/AKT inhibitor, LY294002, blocked the antimitochondrial apoptosis effects of Rosamultin. Inhibition of PI3K/AKT signaling likely also inhibited the activation of the ERK1/2 signaling pathway, thereby inhibiting the antiapoptotic effects of Rosamultin mediated by PI3K/AKT and ERK1/2 signaling pathways. Similarly, LY294002 blocked the antiapoptotic effects of Kaji-ichigoside F1, by directly inhibiting the activation of PI3K/AKT signaling pathway. This therefore inhibited Kaji-ichigoside F1-mediated activation of the ERK1/2 and the associated antimitochondrial apoptotic effects. We also confirmed that Kaji-ichigoside F1 and Rosamultin inhibited mitochondrial apoptosis of vascular endothelial cells induced by hypoxia, via the activation of ERK1/2 signaling pathway. PD98059 blocked the antiapoptotic effects of Kaji-ichigoside F1 on mitochondrial apoptosis, suggesting that the antiapoptotic effect of Kaji-ichigoside F1 was mediated by the activation of the ERK1/2 signaling pathway. Additionally, PD98059 enhanced the antiapoptotic effects of Rosamultin, as a blockade of ERK1/2 signaling was seen to promote activation of the PI3K/AKT signaling pathway, thereby enhancing the effects of Rosamultin.

We carried out the comprehensive evaluation of the protective effects of Kaji-ichigoside F1 and Rosamultin against hypoxia-induced damage in vascular endothelial cells based on TOPSIS (Technique for Order of Preference by Similarity to Ideal Solution). We found that the pharmacodynamics of Rosamultin were better than that of Kaji-ichigoside F1, in terms of protective effects. The differences in pharmacodynamics may be related to differences in the signaling pathways activated. Rosamultin activated PI3K/AKT and ERK1/2 protective signaling pathways in vascular endothelial cells. In contrast, Kaji-ichigoside F1 only activated the ERK1/2 signaling pathway and in doing so, inhibited PI3K/AKT signaling. It is possible that different drug conformations impact binding between drugs and their receptors, resulting in different mechanisms of action that alter pharmacodynamics.

Traditional Chinese medicine (TCM), offering unique medicines for the prevention and treatment of diseases, has been applied in clinics for thousands of years. TCM contains multiple active ingredients. However, due to the complexity and diversity of TCM, its effectiveness continues to be debated. Therefore, for new drug candidates, research that is aimed at identifying the active component or components of herbal medicines is crucial in the drug research process [53–55]. Based on the traditional application of *Potentilla anserina* L. and previous studies on the n-butanol extract [5–7], we isolated Kaji-ichigoside F1 and Rosamultin from the n-butanol extract of *Potentilla anserina* L. and confirmed the antihypoxic activity of the two single compounds. This study elucidated the molecular mechanisms of Kaji-ichigoside F1 and Rosamultin antihypoxic activity and provided a theoretical and experimental basis for further investigation of the antihypoxic effects of *Potentilla anserina* L.

## 5. Conclusion

The results of the present study indicated that Kaji-ichigoside F1 protected vascular endothelial cells against hypoxia-induced mitochondrial apoptosis by activating ERK1/2 signaling, which positively regulated NF-*κ*B signaling and negatively regulated the PI3K/AKT signaling pathway. The antihypoxic mechanism of Rosamultin was related to PI3K/AKT and ERK1/2 and the regulation of NF-*κ*B signaling pathways. Rosamultin protected vascular endothelial cells against hypoxia-induced apoptosis by activating the PI3K/AKT signaling pathway and positively regulating ERK1/2 and NF-*κB* signaling pathways. Our study provided a theoretical and experimental basis for revealing the potential use of *Potentilla anserina* L. as an antihypoxic therapy.

## Data Availability

The data used to support the findings of this study are included within the article.

## Supplementary Materials

Supplementary Materials Fig: drug concentration screening was completed by MTT. (A) Kaji-ichigoside F1 at different concentrations significantly improved cell viability during hypoxia. (B) Rosamultin at different concentrations significantly improved cell viability during hypoxia. The OD values were shown as mean ± SEM (*n* = 6). ^+^*P* < 0.05 vs. normoxia control group; ^∗^*P* < 0.05 vs. hypoxia model group.
