# Supplementary figures and images for: Kaji-Ichigoside F1 and Rosamultin Protect Vascular Endothelial Cells against Hypoxia-Induced Apoptosis via the PI3K/AKT or ERK1/2 Signaling Pathway
Source: Oxid Med Cell Longev. 2020 Apr 12;2020:6837982. doi: 10.1155/2020/6837982 (PMC7153006; doi:10.1155/2020/6837982)

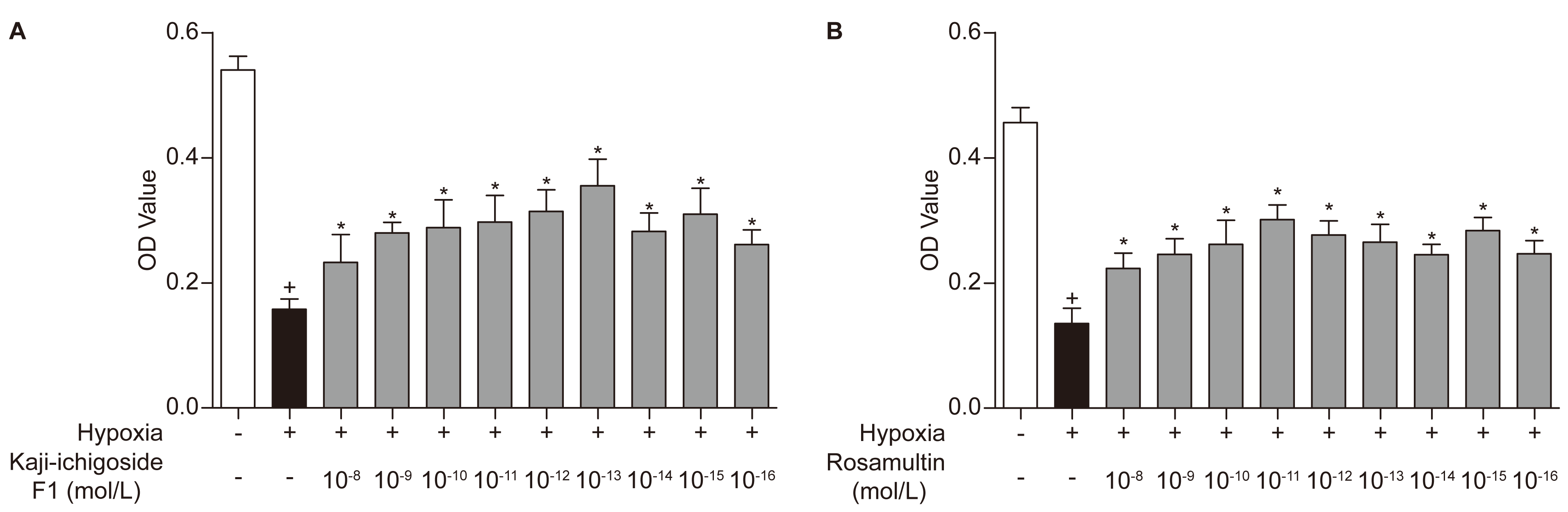

Supplement: Supplementary Materials — Fig: drug concentration screening was completed by MTT. (A) Kaji-ichigoside F1 at different concentrations significantly improved cell viability during hypoxia. (B) Rosamultin at different concentrations significantly improved cell viability during hypoxia. The OD values were shown as mean ± SEM (n = 6). +P < 0.05 vs. normoxia control group; ∗P < 0.05 vs. hypoxia model group. [file 6837982.f1.tif]
